# Toward Robust Characterization of Dynamic Binding Pockets: Lessons from the HBV Capsid Assembly Modulator Site

**DOI:** 10.64898/2026.08.06.743403

**Published:** 2026-08-10

**Authors:** Carolina Pérez-Segura, Liam W. Scott, Adam Zlotnick, Jodi A. Hadden-Perilla

## Abstract

Protein function often depends on ligand binding pockets that fluctuate among conformational states, altering their size, shape, topology, and accessibility, yet quantitative comparison of these dynamic cavities remains challenging because their boundaries are often inherently ambiguous. The *measure volinterior* algorithm uses fuzzy-boundary detection to characterize enclosed molecular spaces; here, the hepatitis B virus (HBV) capsid assembly modulator (CAM) binding site is used as a model system to develop and validate a practical workflow for applying the method to dynamic protein binding pockets. The resulting methodology provides practical guidance for parameter selection and evaluation, establishes a standardized protocol for quantitative characterization of the HBV CAM pocket, and demonstrates robust, reproducible performance across conformational ensembles derived from molecular dynamics (MD) simulations. More broadly, this work provides a reproducible strategy for adapting *measure volinterior* to other dynamic binding pockets, enabling consistent comparison of pocket geometry among independent structural studies.

## Full Text Availability

The license terms selected by the author(s) for this preprint version do not permit archiving in PMC. The full text is available from the preprint server.

